# what do reactive astrocytes (really) do?

**DOI:** 10.1002/alz.088060

**Published:** 2025-01-09

**Authors:** Shane A. Liddelow

**Affiliations:** ^1^ NYU Grossman School of Medicine, New York, NY, USA; NYU, New York City, NY USA

## Abstract

**Background:**

Astrocytes, a major glial cell in the central nervous system (CNS), can become reactive in response to inflammation or injury, and release toxic factors that kill specific subtypes of neurons. Over the past several decades, many groups report that reactive astrocytes are present in the brains of patients with Alzheimer’s disease, as well as several other neurodegenerative diseases. In addition, reactive astrocyte sub‐types most associated with these diseases are now reported to be present during CNS cancers of several types. Given the active role some of these reactive astrocyte sub‐states in these contexts, it suggests that targeting astrocyte reactivity may be a novel therapeutic strategy for multiple disorders. Here we explore the link between astrocyte reactivity and brain cancer, and found that some brain tumors can induce a similar form of reactive astrocytes that promote tumor growth and invasion.

**Method:**

We used a combination of single cell/nuclei RNAseq, ATACseq, spatial transcriptomics, protein and lipid analyses, in both rodent primary and human iPSC‐derived astrocytes, as well as behavioral assays in mice that lack key components of astrocyte reactivity pathways.

**Result:**

Multiple sub‐states of reactive astrocytes first described in the context of acute inflammatory responses, or chronic neurodegenerative disease, are present in the brains of rodent models of cancer. This includes oligodendroglioma and melanoma metastases.

**Conclusion:**

Astrocytes while being very heterogeneous at the measurable 'omics level and with respect to functional output WITHIN an individual disease, can have sub‐states that are homogenous across multiple disease states. This means that functional/dysfunctional outcomes learning in one model may be translatable to further out understanding of disease pathogenesis in another model. This has been historically reported in the comparison of (say) spinal cord injury and stroke ‐ both of which produce a proliferative border/scar formed by reactive astrocytes, but is now appreciated at multiple pro‐inflammatory reactive states including neurotoxic reactive astrocytes, interferon‐responsive reactive astrocytes, and likely many more.

